# Repeated subretinal surgery and removal of subretinal decalin is well tolerated - evidence from a porcine model

**DOI:** 10.1007/s00417-017-3704-z

**Published:** 2017-06-12

**Authors:** Nina Buus Sørensen, Kristian Klemp, Troels Wesenberg Kjær, Steffen Heegaard, Morten la Cour, Jens Folke Kiilgaard

**Affiliations:** 1 Department of Ophthalmology, Copenhagen University Hospital, Rigshospitalet, Copenhagen, Denmark; 2 Department of Neurology, Zealand University Hospital, Roskilde, Denmark; 3 Department of Pathology, Copenhagen University Hospital, Rigshospitalet, Copenhagen, Denmark

**Keywords:** Robotic surgery, Retinal pigment epithelium, RPE, Retinal detachment, Subretinal space

## Abstract

**Purpose:**

Subretinal perfluorocarbon liquid (PFCL) is a serious complication that can occur after retinal detachment repair. It is possible to remove the PFCL surgically, but retinal damage related to the procedure is unknown. Also, increasing interest in subretinal treatment makes it relevant to examine the functional and morphological consequences of repeated subretinal manipulation. We hypothesized that PFCL in a porcine model can be injected in the subretinal space and removed with minimal effect on retinal structure and function.

**Methods:**

The left eyes of ten healthy three-month-old female domestic pigs were included. Multifocal electroretinograms (mfERG) were recorded before surgery. Following vitrectomy, a PFCL bleb (decalin) was injected subretinally using a 41G cannula. After 14 days the decalin was removed through a 41G cannula in combination with a 2 ml syringe and an intermediate flexible tube. Two weeks after removal, a control mfERG was recorded, the pigs were enucleated and sacrificed and eyes were examined histologically. All statistics were carried out with a paired t-test in SAS Enterprise Guide 7.1® (SAS Institute Inc., Cary, NC, USA).

**Results:**

There was no significant difference in mfERG amplitude ratio (left/right eye) between baseline and recordings two weeks after removal of decalin (P1 (M = 0.26, SD = 0.80, *p* = 0.39), second order kernel (M = −0.18, SD = 0.86, *p* = 0.57), Direct Response (M = 0.39, SD = 0.61, *p* = 0.12) or Induced Component (M = −0.03, SD = 0.40, *p* = 0.80)). Histologically, the photoreceptor outer segments were minimally affected. Otherwise the retina was normal 14 days after removal of decalin. In four pigs the subretinal decalin displaced inferiorly and was no longer accessible for removal.

**Conclusion:**

Subretinal decalin can be removed within 14 days without lasting retinal damage. Decalin is a heavy liquid where the risk of displacement is high. Future studies using PFCLs to control duration of an experimental retinal separation should focus on PFCLs that are isodense to the vitreus body.

## Introduction

A well-known complication to intraocular surgery in humans is the deposition of subretinal perfluorocarbon liquids (PFCLs) [1–3]. Decalin is a PFCL commonly used for retinal stabilization in vitreoretinal surgery [4]. Once decalin enters the subretinal space, it remains there without visible change in size [3]. Subretinal decalin can be removed by advancing the droplets toward a retinotomy and aspirating it with a fluid needle and a conventional oil pump [2]. It can also be removed by manual aspiration with a blunt-tipped 21-gauge needle [1]. It is unclear how the removal of decalin affects retinal structure and function. Furthermore, the toxicity of intraocular decalin has been discussed. In rabbits, subretinal decalin induced severe retinal vacuole formation, degeneration, necrosis and advanced damage to the photoreceptor nuclei and outer and inner segments [5]. In vitro, the vitality of human RPE cells incubated with decalin is decreased, but the proliferation of surviving cells is normal once decalin is removed [6]. In humans, the presence of short-term subretinal decalin outside the macula does not seem to affect visual acuity or anatomic success after retinal detachment repair [3, 7]. It is, therefore, relevant to examine toxic and mechanical damage in relation to subretinal decalin and repeated subretinal surgery.

The increasing demands for subretinal procedures, such as cellular transplants, gene therapy and long-acting drugs, makes it relevant to investigate the consequences of re-entry into the subretinal space in experimental models. An experimental model where subretinal fluid can be removed when desired would also be useful for studies of retinal detachment. One problem in retinal detachment studies has been that the duration of separation has been difficult to control. Short-term retinal separation can be obtained with ringer lactate, but the retina reattaches spontaneously [8]. Long-term retinal separation without reattachment has been obtained with Healon [9]. Surgical removal of Healon is challenging, as it requires a high flow to shift qualities from cohesive to dispersive, the viscosity necessary for its removal with a syringe. Such a high flow would probably damage the retina. PFCLs such as decalin are, therefore, interesting as an alternative space-occupying substance to induce controlled retinal separation. The low viscosity allows easy injection and aspiration through microsurgical instruments [4, 7]. Other experimental models that would benefit from controlled retinal separation are studies of RPE atrophy in dry AMD and of subretinal transplants. Earlier studies have emphasized the importance of separation between photoreceptors and Bruch’s membrane as gliosis occurs in areas devoid of retinal pigment epithelial (RPE) cells [10–14].

In this study we evaluated the structural and functional consequences of repeated subretinal surgery and subretinal decalin deposition with histology and mfERG in a porcine model. Furthermore, we explored techniques for subretinal injection and removal of decalin.

## Methods

The research protocol complied with the ARVO Statement on the use of animals in ophthalmic and vision research and was approved by the Danish Animal Experiments Inspectorate (Number 2012–15–2934-00151). A veterinarian supervised all animal procedures. A power analysis was performed prior to the study [15] with SD = Standard deviation (from baseline in previous studies) = 2.7 nV/deg^2^, Z^α^ = 1.96 (from Z table) at type I error of 5%, Z^β^ = 2.33 (from Z table) at 99% power and d = effect size = difference between mean values = 5%. This gave sample size = SD^2^ (Z^α^ + Z^β^)^2^/d^2^ = 2.7^2^(1.96 + 2.33)^2^/5^2^ = 5.37 animals ≈ six animals. The study included a total of ten healthy left eyes of three-month-old female domestic pigs of Danish Landrace⁄Duroc⁄Hampshire⁄Yorkshire breed weighing approximately 30 kg. Six pigs completed the study as planned. One pig was excluded and sacrificed due to stomach infection and high fever, and not responding to antibiotics. In four pigs, the decalin bleb sank inferiorly and did not remain in the visual streak at the time for removal of the decalin. Data from these pigs were included with the intention to evaluate the effect of short-term (< 1 week) detachment of the visual streak with decalin.

### Anesthesia

For a period of 18 h prior to anesthesia, animals were fasted but had free access to water. The animals were preanesthetized with Zoletil, followed by right pupil dilation to >8 mm with a combination of topical 0.4% benoxinate hydrochloride (oxybuprocaine; SAD, Copenhagen, Denmark), 10% phenylephrine hydrochloride (metaoxedrine chloride; SAD), 0.5% tropicamide (Mydriacyl; Alcon, Heinaut, Belgium) and 1% atropine sulfate (SAD). The pigs were endotracheally intubated and artificially ventilated. Artificial ventilation was supplied with 0.5 l/min 100% oxygen and 2.5 l/min atmospheric air. The stroke volume (10 ml/kg) and respiratory frequency (16/min) were maintained at a constant rate. The pigs were anesthetized by administration of intra-venous Propofol 15 mg/kg (B. Braun Melsungen, 10 mg/ml). The pigs were kept hydrated via NaCl i.v. (Fresenius Kabi 9 mg/ml) and body temperature was maintained at 38–39 °C. Heart rate, EKG, carbon dioxide and oxygen saturation levels were monitored.

### Baseline mfERG and surgical procedures

In the fully anesthetized animals a baseline mfERG was obtained. After completion of the mfERG recordings, three sclerotomies were obtained at 10, 2, and 5 o’clock, 2 mm posterior to the corneal limbus. The infusion line was secured inferiorly with Ringer Lactate (SAD, Copenhagen, Denmark) and a standard three-port vitrectomy including removal of the posterior hyaloid was performed using a 21 G vitrectomy probe (Karl Storz GmbH, Tuttlingen, Germany). Hereafter, decalin (Eftiar® Decalin, Perfluoro-Decalin, DORC, Zuidland, The Netherlands) was injected subretinally close to a retinal vessel above the optic nerve in the area of the visual streak with a 41G/0.1 mm BSS injection needle (REF 1270.01, DJ Instrumenter, Denmark) to produce a prolonged retinal detachment (bleb). The detachment was enlarged to the desired size by regulating the amount of decalin injected (200–300 μL). Sclera and conjunctiva were sutured with 7–0 coated Vicryl (Ethicon, Inc., Norderstedt, Germany), and topical application of 1% chloramphenicol ointment (Kloramfenikol “DAK”, Nycomed, Roskilde, Denmark) was applied to the eye at the end of the procedure. Two weeks (eight days in one pig due to practical circumstances) after initial surgery, access to the retina was obtained with the same methods previously described and decalin was removed manually using the 41G BSS needle, an intermediate piece (plastic tube from the vitrectomy water infusion set) and a 2 ml syringe.

### Follow-up procedure

Two weeks post decalin removal, the animals were re-anesthetized with the addition of a neuromuscular blocker to avoid eye movement; 4 mg cisatracurium i.v. (Nimbex®, GlaxoSmithKline, Brøndby, Denmark). MfERG recordings were conducted in an electrically shielded room under standardized lighting conditions and dilated eyes were adapted to room light for 15 min. A Burian-Allen bipolar contact lens electrode (VERIS™ Infrared (IR) Illuminating Electrode; EDI Inc., Redwood, CA, USA) was placed on the cornea with a gel (Viscotears®, Novartis, Copenhagen, Denmark) as contact fluid. A reference electrode was placed behind the ear and the animal and all electrical equipment were electrically grounded. The mfERG equipment allowed continuous infrared (IR) fundus monitoring during recordings to detect eye movement. In all animals, after indirect ophthalmoscopy, color fundus pictures were acquired. An injection of fentanyl-hammeln (Hammeln pharmaceuticals GmbH, 50 μg/ml) 5 μg/kg i.v. was given and the left eye was enucleated. The anesthetized pigs were then euthanized using 1 ml/kg Pentobarbital 200 mg/ml Lidocainhydrochlorid 20 mg/ml i.v. (Glostrup Pharmacy, Denmark).

### MfERG settings

Multifocal electroretinograms were recorded using a VERIS™ Multifocal System with VERIS 6.0.8 software (EDI, Inc., Redwood, CA, USA). The system included a FMS III stimulator, refractor, eye- and IR fundus monitoring. The conventional mfERG stimulation consisted of one frame that underwent a pseudorandom m-sequence of flash or dark frames. In the global-flash mfERG, four frames are presented in a row: one m-sequence frame, one full-field dark frame, one full-field flash frame and another full-field dark frame. A black-and-white 103 unscaled hexagon stimulus pattern was used, at a frame rate of 75 Hz, with 16 samples per frame. The m-exponent was 15 and the durations of recordings were 7.17 (conventional) and 14.34 (global-flash) minutes, respectively.

### MfERG analysis

The IR fundus picture with the stimulus grid from the VERIS™ system was aligned with a corresponding fundus picture using Adobe Photoshop CC - Adobe®. The mfERG traces could thereby be superimposed on the digital fundus photograph, securing the exact corresponding anatomical localization of each hexagon [16]. The area of interest was easy to identify on the fundus photography, and, by using the aligned photos, it was possible to identify the mfERG hexagons corresponding to the lesion. Responses that were obtained entirely within areas of reattached retinas were summed and responses from the corresponding area of the healthy eye were used as control. This reduced variation due to anesthesia and the individual, and therefore stabilized the variance.

### Histology

**Fig. 1:**
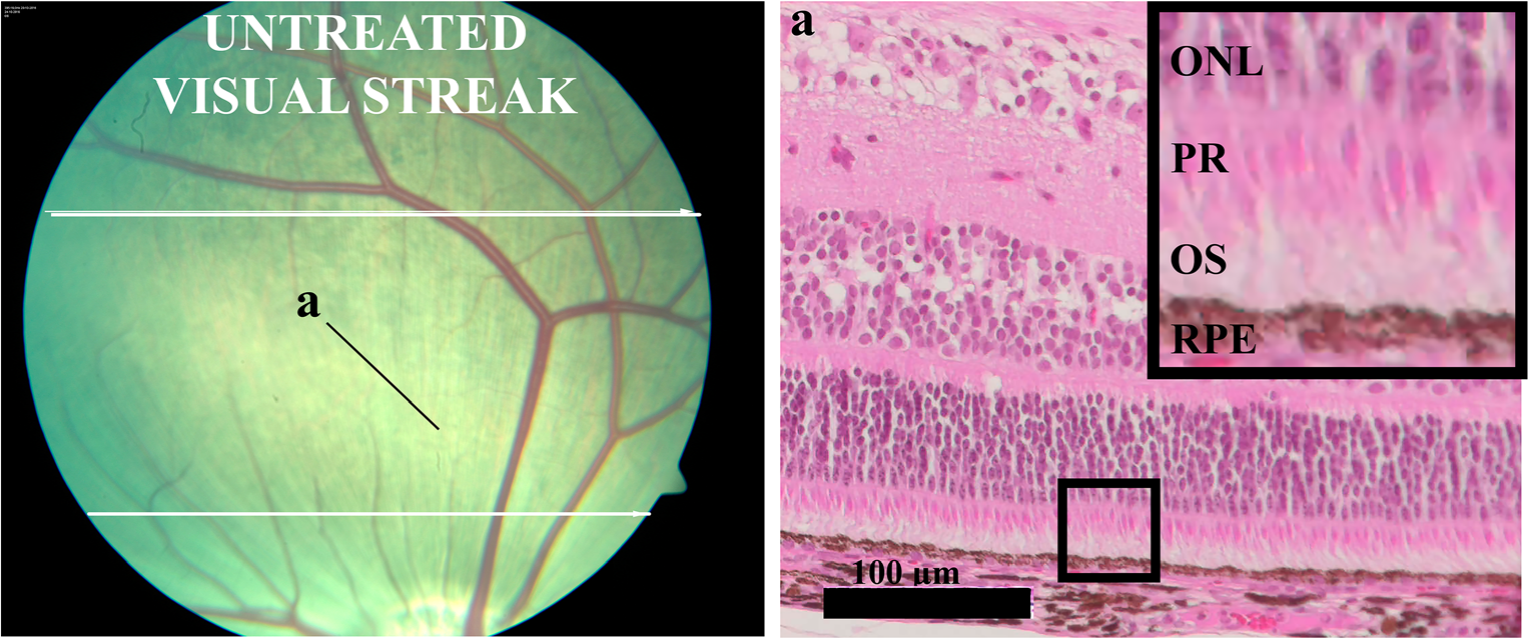
The untreated retina in the visual streak (horizontal zone between the white lines in the fundus photograph) with the evaluated cell layers outer nuclear layer (ONL), photoreceptor layer (PR), photoreceptor outer segments (OS) and retinal pigment epithelium (RPE). The small quadrangle in the lower magnification histologic image represents the area magnified in the large quadrangle to the right

The anterior segment and lens of the enucleated eye were removed, and the posterior segment was fixed in paraformaldehyde 4%. Segments containing the optic disc and the surgical lesion were identified, isolated and embedded in paraffin, according to standard procedures. Sections of 5 μm were taken through the lesion and examined with a light microscope (LM), (Axioplan 2, Carl Zeiss, Jena, Germany). Digital images were obtained with an Axiocam HRC (Carl Zeiss), as described previously [10]. The histological findings were mapped onto the fundus photographs using retinal vessels, vessel crossings and the optic nerve as “landmarks” [13]. All histological slides were thoroughly examined and graded according to appearance of outer nuclear layer, photoreceptor layer (PR), photoreceptor outer segments (OS) and retinal pigment epithelium (RPE) (Fig. 1). Each category was graded ordinally where 0 = normal conditions, 1 = layer affected, 2 = layer degenerated, 3 = maximal damage with no retinal organization. The specimen were also examined for remnants of decalin and choroidal neovascularization (CNV), where 0 = not present and 1 = present. Ganglion cells were counted in a treated and an untreated area in the visual streak in the same histological specimen. Three specimens per pig were investigated. From each histological specimen, two digital images were obtained, one from the treated and one from the untreated area. On the images a zone of 500 μm was marked and further magnified so the zone of 500 μm filled out the computer screen. Ganglion cells were counted within the 500 μm zone using Fiji ImageJ 1.49. Only cells with a clearly defined cell wall were counted, and, in case of doubt, the cell was not counted.

### Data analysis

Means and 95% confidence intervals (mean CI) of amplitude-ratio (Right/Left eye), as well as means and standard deviations (mean ± SD) of implicit time-ratios (Right/Left eye) are given. The ratio of the major amplitudes (P1 and IC) and the number of ganglion cells (treated versus untreated area) were analyzed using a paired t-test in SAS Enterprise Guide 7.1® SAS Institute Inc., Cary, NC, USA.

## Results

### Retinal function is normal 14 days after removal of decalin

**Fig. 2:**
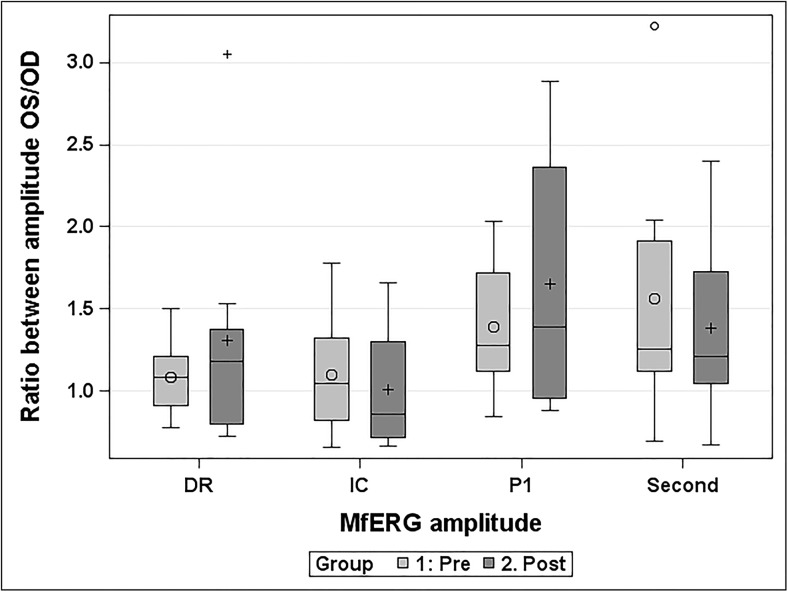
The average ratio between left (OS) and right eye (OD) for the global-flash amplitudes and conventional amplitudes before retinal detachment (pre) and 2 weeks after reattachment (post). o and + within the box = median. Line within the box = average. There were two outliers, presented as o and + in the superior part of the fig. OS = oculus sinister, OD = oculus dexter, DR = direct response (global-flash mfERG amplitude), IC = induced component (global-flash mfERG amplitude), P1 = conventional mfERG amplitude, Second = second order kernel (conventional mfERG amplitude)

Two weeks after the removal of decalin, mfERG amplitudes recorded from reattached retinal areas were normal. There was no significant difference between the pre-surgery and the 14 day-reattachment mfERG ratios between the left and right eye for the conventional first-order P1 amplitude (M = 0.26, SD = 0.80, *p* = 0.39), the conventional second order kernel (M = −0.18, SD = 0.86, *p* = 0.57), the global-flash direct response (DR) (M = 0.39, SD = 0.61, *p* = 0.12) or the global-flash-induced component (IC) (M = −0.03, SD = 0.40, *p* = 0.80) (Fig. 2).

### The retinal condition is unrelated to the presence of decalin

**Fig. 3:**
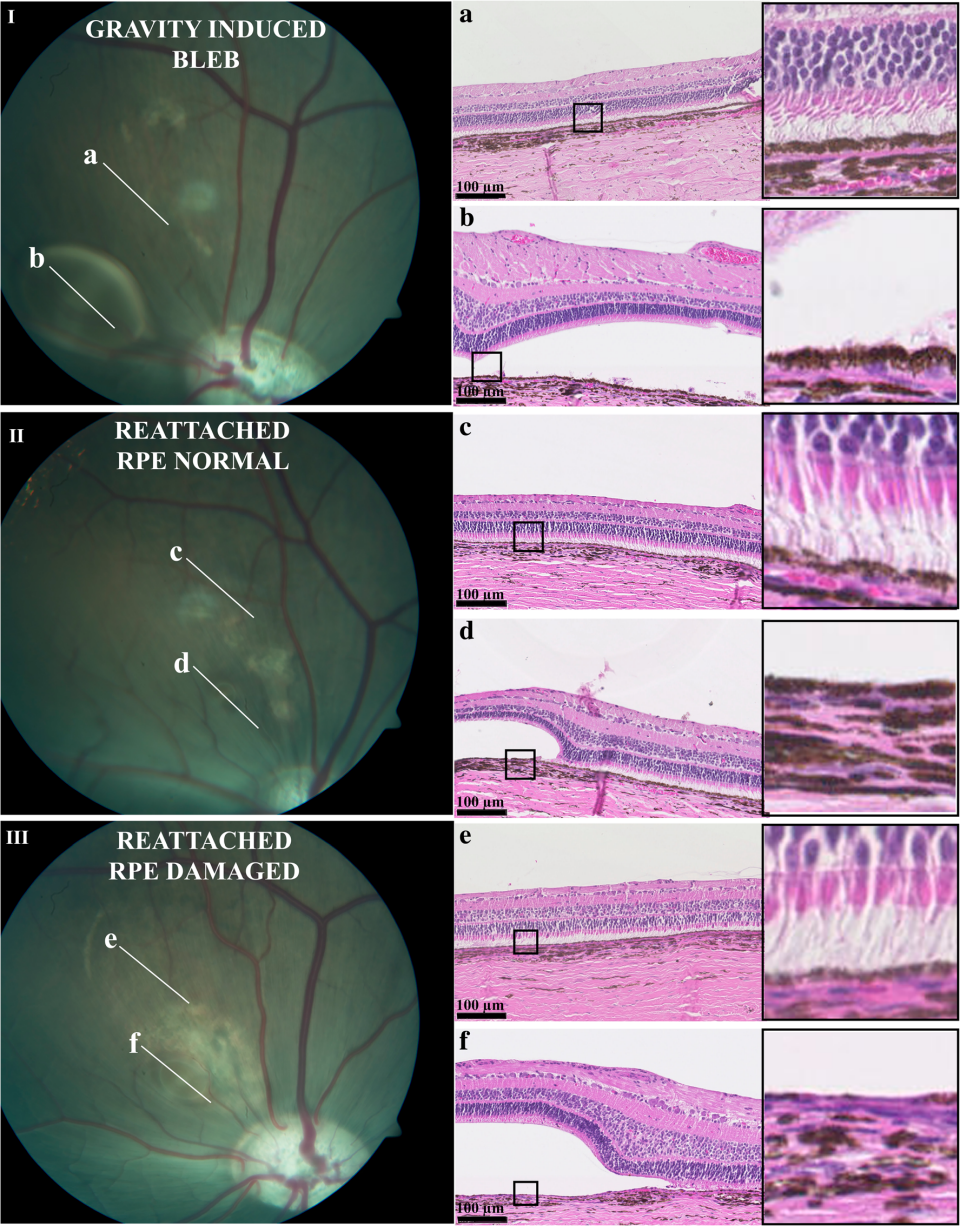
**I**) Gravity has displaced subretinal decalin inferiorly to the visual streak. *a*) A section through the spontaneously reattached area shows normal conditions. *b*) A section through the spontaneously formed bleb shows normal conditions, despite decalin separating the photoreceptors from the RPE cells. Please note the normal RPE layer compared to F, where the bleb was made by injection. **II**) Subretinal decalin removed after 14 days, picture was taken 14 days after reattachment. A small remaining bleb is seen inferior to the reattached area.* c*) A section through the reattached area shows normal histology. *d*) A section through the remaining bleb shows normal histology. **III**) Subretinal decalin was removed 14 days earlier, remnants have gathered into a small inferior bleb. *e*) A section through the reattached area shows thinning of the RPE-layer. *f*) A section through the area with remaining decalin shows absence of the RPE layer. The small quadrangle in the lower magnification histologic image represents the area magnified in the large quadrangle to the right

Six of seven eyes had affected (shortened) photoreceptor outer-segments (graded 1), one of seven eyes had an affected RPE layer (graded 1) and two of seven eyes had a CNV . The variation in retinal damage is presented in Fig. 3. The mean number of ganglion cells in treated areas was 25.43 cells and the mean number in untreated areas was 23.57 cells. There was no significant difference between the treated and the untreated areas regarding number of cells in the ganglion cell layer (*p* = 0.1, CL (−0.47, 5.14)).The fundus photographs were examined for hypopigmented areas as an indication of RPE damage. Corresponding to the retinotomies, localized hypopigmented areas were found in all pigs. In one of the seven pigs, RPE damage covered the full extent of the detached area (Fig. 3).

## Discussion

We found minimal retinal damage after short-term retinal detachment induced by subretinal decalin injection and subsequent removal of decalin. In two of the animals a CNV was seen. The CNV is induced by lesions to the Bruch’s membrane [17]. The CNVs in our study was found in relation to the retinotomies. There is a risk of damaging the Bruch’s membrane when entering the subretinal space in an area with attached retina. For removal of Decalin, this risk is minimal as the distance between the retina and the Bruch’s membrane is larger in the detached area. We, therefore, consider the risk of inducing a CNV in relation to removal of subretinal decalin to be minimal. Consequently, it seems that clinical practice may safely include removal of iatrogenic subretinal decalin within 14 days. In reattached areas the outer and inner retinal function measured with conventional and global-flash mfERG was normal. Histologically the retinal changes in reattached areas were minimal. Thinning of the RPE layer was mainly seen in relation to retinotomies where the decalin was injected, and never in areas with accidental subretinal decalin. This indicates that RPE damage is related to the injection and not the presence of decalin itself. It is possible that the velocity with which the fluid was injected into the subretinal space affected the degree of retinal damage. As the decalin was injected manually, it is likely that the velocity with which the fluid entered the subretinal space varied. This could explain the inter-individual variability in RPE-damage (Fig. 3). Shortening of the photoreceptor outer segments was seen in areas with sustained retinal detachment, but the morphological organization of the retinal layers was surprisingly preserved. In contrast, previous studies using Healon to induce RD demonstrated massive histological changes [18–22]. Perfluorocarbon liquids (PFCLs) differ from Healon in that PFCLs can carry oxygen and carbon dioxide [23]. It is thus possible that some gas-exchange occurs across the subretinal decalin and nourishes the detached retina. Furthermore, contrary to the capillary-free human fovea the porcine visual streak is supplied by capillaries [24]. It is, therefore, likely that the retinal vasculature to some degree supplies the detached porcine retina with oxygen and nutrients, which could explain the preserved retinal architecture. It is also possible that the effect of short-term subretinal decalin is comparable to that of an acute serous detachment in humans, which generally resolves spontaneously with minimal sequela [25].

In humans, short-term intraocular PFCL has also been associated with good functional outcomes [3, 7], whereas prolonged exposure has been associated with RPE atrophy and photoreceptor damage [26–29]. Based on our findings, it is not likely that the harmful consequences of long-term subretinal decalin are due to a toxic effect. Fourteen days after removal of decalin, we found large inter-individual variability in the degree of retinal damage. To explore this variability, we examined blebs that had accidentally formed by the inferior displacement of decalin by gravity. Migration of subretinal decalin has also been observed in humans [29]. Despite decalin in these blebs, we only found shortening of photoreceptor outer segments, otherwise the retina was histologically unaffected. The shortening of photoreceptors resembles the temporary effect seen in short-term retinal detachment with ringer-lactate [8]. Ringer-lactate is non-toxic and the shortening of photoreceptor outersegments is, therefore, thought to be caused by the separation of photoreceptors and RPE-cells, rather than the substance itself. If decalin was toxic, more pronounced retinal damage would be expected, as seen in studies of subretinal indocyanin green (ICG) [30]. One explanation for the retinal damage related to prolonged exposure could be the lack of sufficient nutrients. This corresponds to our finding in detached areas where RPE cells resting directly on the choroid are normal while photoreceptors without contact to the underlying layers are affected. Other studies have suggested that PFCLs form barriers for the normal diffusion and transport of substances, and impedes normal metabolic exchange [5, 31]. Another factor that could cause damage with prolonged exposure is decalin’s specific gravity. A study in rabbits showed that retinal damage in relation to intraocular tamponade with perfluorocarbon liquids was restricted to inferior areas [32]. Several case-reports have, therefore, recommended the removal of accidental subretinal decalin within a short time [1, 29]. It seems that this can safely be done, as we found that repeated surgical entry into the subretinal space minimally affected retinal architecture and function.

**Fig. 4:**
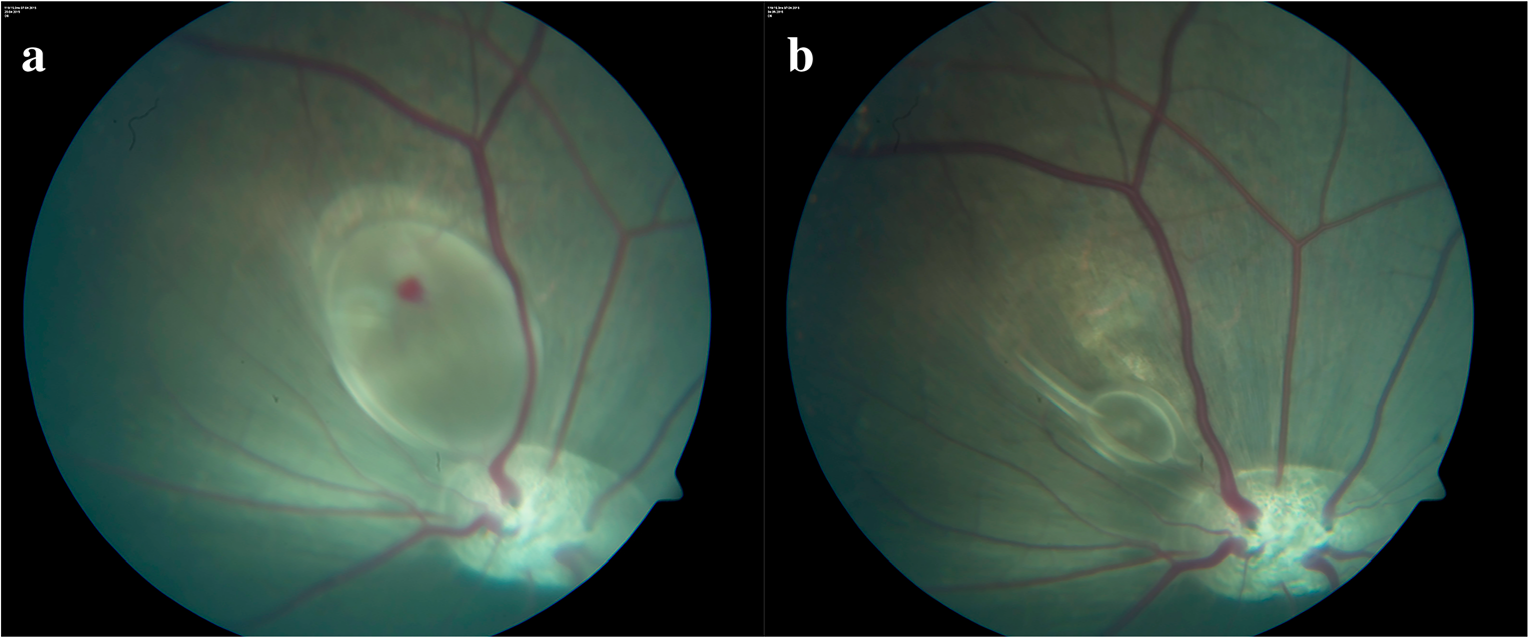
**a** The decalin-filled bleb 14 days after injection. **b** The retina has reattached in most of the area 14 days after removal of decalin. A minor remnant of decalin has gathered into a small bleb inferiorly in the reattached area superior to the optic disc

This study further demonstrates that the duration of experimental retinal detachment can be controlled by surgically removing an injected subretinal fluid. The model offers the possibility to control the duration for which the photoreceptors are not in contact with the RPE. However, one challenge with the model is that a large amount of decalin can be pulled down by gravity and moved away from the area of interest. This is probably due to decalin’s density, which is approximately twice that of water [28]. The model, therefore, needs to be adjusted, and we recommend that future studies focus on low-viscosity PFCLs that are isodense to the corpus vitreum. Technical optimization is also desirable for subretinal surgery. Another challenge we experienced with the model was that, when we entered the injection-area twice, a reflux of decalin from the first injection site took place. Hence, it was impossible to inject larger amounts if there was an accidental withdrawal of the 41G needle from the first injection site. Furthermore, it was difficult to remove all subretinal decalin in one procedure, as the unattached retina captured small remnants of decalin (Fig. 4). Robotic surgery eliminates tremor and may enable better control of injection, flow velocity and volume injected/aspirated. Furthermore, perioperative optical coherence tomography (OCT) would likely lessen the reflux problem, as it would assure the correct positioning of the needle at all times.

## Conclusion

The effect of repeated surgical entry into the subretinal space and short-term subretinal decalin is minimal. We recommend that removal of accidental subretinal decalin within 14 days is considered.
